# Modification of the dextran sodium sulfate model to identify agents that induce or exacerbate inflammatory bowel disease

**DOI:** 10.1080/1547691X.2025.2594800

**Published:** 2025-12-29

**Authors:** Victor J. Johnson, Michael I. Luster, Michael Kashon, Erin M. Quist, Gary R. Burleson, Florence G. Burleson, Dori R. Germolec

**Affiliations:** a Burleson Research Technologies, Inc, Morrisville, NC, USA; b Mountainview Research Analytics, LLC, Morgantown, WV, USA; c Experimental Pathology Laboratories, Inc, Durham, NC, USA; d Division of Translational Toxicology, National Institute of Environmental Health Sciences, NIH, DHHS, Durham, NC, USA

**Keywords:** Irritable bowel disease, inflammatory bowel disease, ulcerative colitis, Crohn’s, immunotoxicity, IBD exacerbation

## Abstract

Exposure to environmental agents has been linked to inflammatory bowel disease (IBD), including ulcerative colitis (UC) and Crohn’s disease (CD) in humans. In these studies, we describe a modification of an experimental model used historically in the pharmaceutical industry to help identify therapies for the treatment of IBD to facilitate its use for identification of environmental agents that have the potential to accelerate, exacerbate, and/or impair recovery from IBD. In this model, female C57BL/6 mice were exposed to low levels of dextran sodium sulfate (DSS) for 7 consecutive days in drinking water to allow for a modest level of colon inflammation and pathology as measured by a battery of clinical, pathological, toxicological endpoints (water consumption, body weights, colon length, body temperature, stool consistency, and hematochezia), and cytokine/chemokine production in the serum and colon. Treatment with DSS for 7 d showed a clear dose-response with 1% DSS producing minimal changes in the colon and 3% DSS inducing severe damage with IBD. A concentration of 2% DSS in drinking water for 7 d was selected for investigating disease recovery and exacerbation by an environmental agent as it induced mild colon inflammation that showed nearly complete resolution within 21 d following cessation of DSS exposure. Cytokine and chemokine profiles showed a Type 1 predominant immune response in the colon and serum that is consistent with inflammation observed in human IBD. The model was used to determine the impact of administration of a high salt diet (HSD) on DSS IBD progression, severity, and recovery. While administration of HSD by itself had no effect on indicators of colon damage or inflammation, co-administration of HSD with DSS, produced marked exacerbation and persistence of disease supporting the potential of the model for identifying environmental agents that can affect IBD.

## Introduction

Inflammatory bowel diseases (IBD), including ulcerative colitis (UC) and Crohn’s disease (CD), have a complex and multifactorial etiology affecting approximately 3 million individuals in the USA, with an associated health care burden of $8.5 billion annually (CDC 2025). It is marked by chronic relapsing inflammation of the gastrointestinal (GI) tract involving multiple genetic factors, immune responses, and environmental factors; the latter including the gut microbiome, lifestyle, diet, stress, and chemical exposure (Zhang and Li 2014; Abegunde et al. 2016). The disease is characterized by mucosal injury which can involve the entire colon mucosa and sometimes other parts of the GI tract. Therapeutics are a common cause of new-onset colitis (Hamdeh et al. 2021), with non-steroidal anti-inflammatory drugs (NDAID) estimated to be responsible for 10% of newly diagnosed cases (Tanner and Raghunath 1988; Hamdeh et al. (2021). In addition, NSAID use has been associated with clinical relapse in IBD patients that were in remission (Kvasnovsky et al. 2015), suggesting that drugs can exacerbate existing IBD. Experimental and epidemiological evidence suggests certain environmental chemicals, many of them so called ‘forever’ chemicals, can also initiate or influence the progression of colitis. Sanmarco et al. (2022) utilized an integrated systems approach that combined data from high-through-put biochemical and cell-based assays from the EPA ToxCast database with data generated from zebrafish and murine IBD models and machine learning to identify chemicals that either modulate inflammatory mediators, the peroxisome proliferator-activated receptor (PPAR), or the aryl hydrocarbon receptor (AHR). An example of these efforts is the herbicide propyzamide which was shown to target the AHR-NF-κB-C/EBPβ signaling axis in T-cells and dendritic cells resulting in exacerbated pathology in the zebrafish model of 2,4,6-trinitrobenzenesulfonic acid (TNBS)-induced intestinal inflammation. Triclocarban (Yang et al. 2020), a widely used antimicrobial ingredient, as well as aluminum (Pineton de Chambrun et al. 2014), exacerbate gut inflammation in mouse models for IBD. There are also examples in epidemiological literature supporting environmental exposures and IBD in humans. For example, Ananthakrishnan et al. (2011) reported that an increase in the density of pollutant emission by 1-log was associated with a 40% increase in the rate of hospitalizations for both UC and CD (incidence RR = 1.40; 95%CI: 1.31–1.50), but details of the pollutants were not identified. In a study of individuals highly exposed to perfluorooctanoic acid (PFOA) from the mid-Ohio valley (*n* = 32,000), the relative risk of UC was increased by quartile of cumulative PFOA exposure (1.00, 1.76, 2.73, 2.86; test for linear trend *p* = 0.0001) (Steenland et al. 2013). In a more recent study, PFOA serum levels in UC patients were found to be 38% higher than in the control population (Fart et al. 2021). In a study using data from NHANES, higher 4-tert-octylphenol levels were significantly associated with UC (de Silva et al. 2017). These epidemiological studies provide substantial support for a link between environmental exposures to chemicals and IBD in humans.

Experimental animal models have been developed to study the pathobiology of IBD as well as to help identify potential therapeutics. These include mouse or rat models that employ either adoptive T-cell transfer, genetic modification (i.e. transgenic or knockouts) or chemical induction (Valatas et al. 2013; Baydi et al. 2021). Regarding the latter, groups of rats or mice are commonly treated with either dextran sodium sulfate (DSS) or TNBS, to produce colitis. When the model is used in combination with therapeutics information on potential efficacy as wells as unintended toxicity can be obtained (Sha et al. 2013; Chassaing et al. 2014). Unfortunately, there are no widely accepted models that can identify environmental agents that may induce or, more likely, exacerbate IBD. In this study, we adapted the DSS mouse model, a chemical-induced model for IBD, that allows for induction of a low to moderate level of gut pathobiology that is amenable to alteration of disease by environmental agents. Improvements in the study design included assessment of disease severity and recovery at levels of inflammation and damage the allowed for exacerbation of disease and delay in recovery. We also characterized the inflammation in the colon using multiplex cytokine and chemokine analyses which further current understanding of pathogenesis, potential biomarkers, and translation to humans. The utility of this model was demonstrated in DSS treated mice concurrently fed a high-salt diet (HSD).

## Methods

### Experimental animals

This work was conducted at Burleson Research Technologies, Inc. (BRT, Morrisville, NC), an AAALAC Accredited facility with PHS Assurance. All animal work was conducted under an approved Institutional Animal Care and Use Committee protocol (NIEHSO 20180613) in accordance with the NIH/NRC Guide for the Care and Use of Laboratory Animals, 8th Edition. Female C57BL/6N mice were obtained from Taconic Biosciences (Hudson, NY), at approximately 7–8 weeks-of-age. The animals were maintained on a 12-h light/dark cycle at 20.5–23.9°C and 35–65% humidity and received NTP-2000 diet (Ziegler Brothers, Inc, Gardner, PA) and tap water *ad libitum*. All mice were provided Crink-l’Nest^™^ (The Andersons, Maumee, OH) as nesting material and enrichment. Mice were randomized to treatment groups (*N* = 10/group) by bodyweight (±20% of mean body weight) and identified by subcutaneous radio frequency implantable device (RFID) implants (Unified Information Devices, Inc., Lake Villa, IL). The mice were housed 5/cage in individually-ventilated cages (Tecniplast, West Chester, PA). Only animals that were healthy prior to the start of treatment were included in these studies. Once treatments commenced, no animals were excluded from the study. At scheduled necropsy, animals were humanely euthanized by CO_2_ inhalation using 100% CO_2_ introduced at 3.65 L/min into a 7.3 L chamber to displace 50% of the atmosphere per minute until breathing ceased and no pedal reflex was observed. Exsanguination and severing the diaphragm were used for confirmation of death.

### Treatment and study design

**Figure 1.**
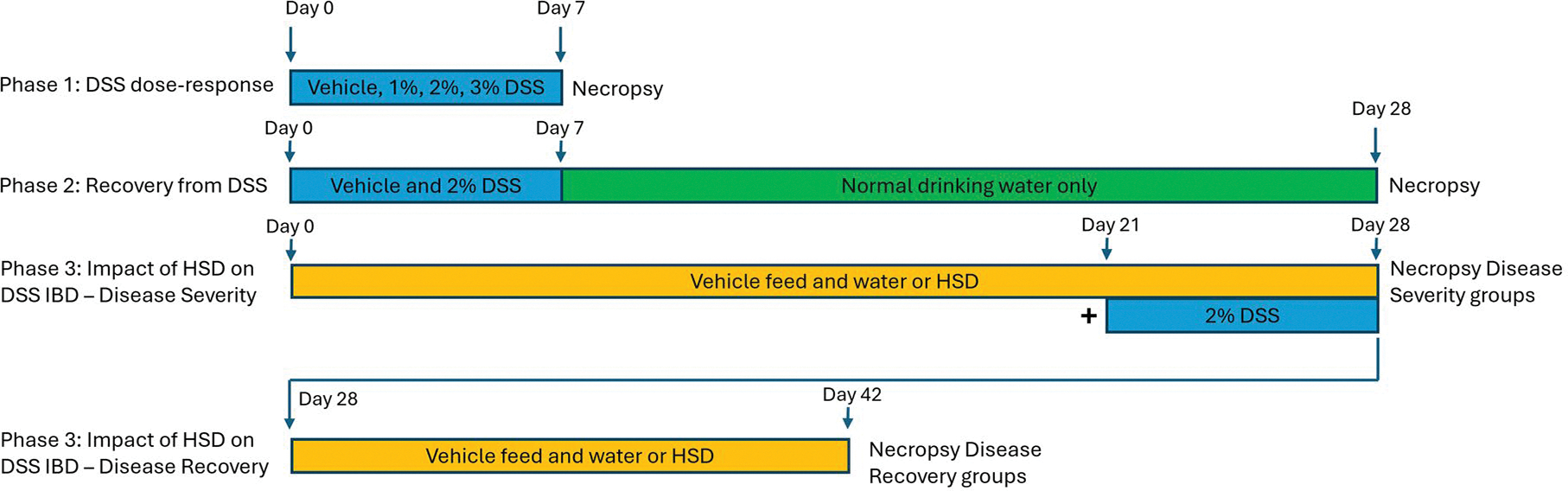
Experimental design is based upon three phases; Phase 1 was a dose-response to determine a concentration of DSS that provides a low-to-moderate level of colon inflammation, Phase 2 established the recovery timeline following cessation of DSS treatment, and Phase 3 was a proof of concept study to demonstrate that exogenous agents could impact disease severity and recovery.

No aspect of these studies was blinded to the staff. Mice were administered DSS at concentrations of 0%, 1%, 2%, or 3% in drinking water for 7 consecutive days. During the period of DSS treatment, drinking water solutions were changed and water consumption was recorded daily. Drinking water was changed and consumption recorded at least twice per week for all other periods. The HSD consisted of NTP-2000 feed supplemented with 4% NaCl prior to pelleting (Zeigler Brothers) in addition to 1% NaCl in the drinking water (Miranda et al. 2018). For groups receiving the HSD, NaCl-containing feed and water were provided *ad libitum* for the duration of the study. The study design used the multi-phase approach provided in Figure 1. These studies required a total of 170 mice.

- Phase 1 study investigated the dose-response for DSS IBD. Groups of mice (10/DSS treatment group) were administered DSS (0%, 1%, 2%, or 3%) in drinking water *ad libitum* for 7 d. Clinical observations, bodyweights and data for disease activity index (DAI) scores were collected daily (Days 0–6) followed by terminal necropsy and sample collection performed on Day 7.
- Phase 2 study investigated the recovery of the GI tract following removal of DSS from the drinking water. DSS (2% in drinking water; selected based on the dose–response performed in Phase 1) was administered to mice for 7 d (Days 0–6) following by normal drinking water until scheduled terminal necropsy. Clinical observations, bodyweights and data for DAI scores were collected daily. Groups of DSS-treated mice (10/necropsy timepoint) were necropsied and samples collected on Days 7, 14, 21, and 28. To reduce the number of animals used for this phase, a single vehicle group (10 mice) was used as comparison to all timepoints and was necropsied on Day 28.
- Phase 3 study investigated the impact of HSD on DSS-induced IBD severity and recovery. Animals were provided with NTP-2000 diet with normal tap water or HSD feed and HSD water throughout the study (Days 0–42). DSS (2% in drinking water) was administered on Days 21–28 by adding it to the normal tap water or HSD water as appropriate. Groups of animals (vehicle, DSS only, HSD only, and HSD + DSS; 10/treatment/timepoint) were necropsied and samples collected on Days 28 and 42 to determine the impact of HSD on DSS IBD severity and recovery, respectively.

### General endpoints

Animals were observed twice daily for signs of toxicity. Detailed clinical observations and cage assessments were conducted at least once per week prior to treatment initiation and daily following the start of DSS administration to assess hydration status including changes in body weight, stool consistency, body temperature (by touch), and the presence of blood in fecal matter. Food and water consumption were measured for each cage on a weekly basis. During DSS treatment, drinking water consumption was measured daily.

### Terminal sample collection

Prior to necropsy mice were euthanized with CO_2_ and a maximum amount of blood collected by cardiac puncture or from the inferior vena cava of each animal into a serum collection tube. The blood was allowed to clot at room temperature for 30–60 min. The tubes were centrifuged at approximately 1300 × *g* at room temperature for ~ 10 min and serum was collected and stored at ≤−70°C until evaluated for cytokine/chemokine levels. The spleen and thymus were weighed and discarded without further evaluation. The large intestine from cecum to rectum was removed, separated from the small intestine at the cecum, and processed as follows.

### Preparation of colon

The colon was gently straightened on a sheet of dental wax to prevent wicking moisture and contraction of tissue, taking care not to stretch the tissue, and the length was recorded. The cecum was removed (discarded) and the colon bisected transversely. The proximal half of the colon was gently flushed using cold PBS Complete buffer (1X PBS supplemented with 2 mM Mg^2+^, 25 U/mL benzonase endonuclease, and protease inhibitors [Complete^™^ Mini, EDTA-free; Roche Diagnostics, Indianapolis, IN]), followed by homogenization in 1X PBS Complete. After disruption/homogenization, samples were incubated on ice for 5 min to allow sufficient time for the endonucleases to reduce the viscosity of the samples. Supernatants were obtained by centrifugation at 10,000 × *g* for 10 min at 4°C and stored at ≤−70°C until analyzed for cytokine/chemokine levels.

The distal half of colon was flushed with 10% neutral buffered formalin (NBF) to remove contents and preserved for histopathology. Swiss Rolls of the distal colon were prepared by carefully wrapping them around a toothpick presoaked in 1X PBS to create concentric rolls. The Swiss Rolls were placed into a histology cassette and immersed in 10% NBF for 24 ± 2 h. Preserved specimens were transferred to Experimental Pathology Laboratories, Inc. (EPL^®^; Durham, NC) where they were placed in 70% ethanol and stored at room temperature until processed for histology. Individual sections were stained with Hematoxylin and Eosin (H&E) for assessment of inflammation and damage and periodic acid–Schiff (PAS) for assessment of mucus and goblet cells.

### Disease activity index (DAI)

The DAI is commonly used to evaluate colitis severity in experimental animals (Cooper et al. 1993). Numeric values of 0–4 were assigned for body weight loss, stool consistency, and the presence of blood in the feces and the total DAI score (range = 0–12), was calculated as the sum of each measure (Table 1). Mice were individually placed in an empty cage for up to 5 min and 3–5 fecal pellets were collected. Stool consistency was scored for individual mice according to the following criteria: normal stool (score = 0), loose stool (score = 2), and diarrhea (score = 4). To determine the presence of blood in feces, fecal matter was homogenized in 1X PBS, luminol reagent added (BLUESTAR^®^ Forensic tablets; BLUESTAR USA, Greer, SC) and full spectrum light production was measured using a Spectramax^®^ i3 luminometer running Softmax^®^ Pro v6.5 (Molecular Devices, LLC., San Jose, CA). Light production less than three standard deviations greater than the mean of the vehicle control samples was considered negative for blood (score = 0), greater than or equal to three standard deviations above the mean of the vehicle samples was a positive reading (score = 2), and gross bleeding observed on the feces at time of collection and/or from the anus received a score of 4.

### Histology scoring

Histopathology services were contracted to EPL^®^, Inc. The severity of colitis was determined histologically in a blinded fashion by examination of H&E- and PAS-stained slides and scoring the extent of inflammatory infiltration and tissue damage. Brief descriptions of the graded histological features are presented in Table 2. The grading scale was from 0 to 4 for each histology endpoint (0 = no lesion/within normal limits; 1 = up to 10%of the tissue affected; 2 = 10–25% of the tissue affected; 3 = 25–50% of the tissue affected; 4 = > 50% of the tissue affected). A total of 3–4 representative fields (10X objective) in each sample were examined to derive the score. The group average severity scores were calculated for each endpoint measured. Colon length was also recorded separately as an additional indicator of inflammation and colon damage.

### Multiplex cytokine/chemokine analyses

Colon homogenates and serum were analyzed for selected cytokines and chemokines shown in Table 3 using MSD V-plex multiplex technology (Meso Scale Diagnostics, LLC. [MSD]; Rockville, MD). The assays were performed according to the manufacturer’s instructions. Data were collected with an MSD QuickPlex SQ120 using Discovery Workbench 4.0.12.

### Data and statistical analysis

Calculation of group means and standard errors (SEM) were performed using Microsoft Excel and/or GraphPad Prism version 6 (GraphPad Software, La Jolla, CA). Statistical analyses were performed using JMP version 17.2 (SAS Institute, Cary, NC). Continuous variables such as body weights and cytokine levels in Phase 1 and Phase 2 studies were assessed using one-way analysis of variance (ANOVA) to examine dose-response and time course relationships, respectively. Phase 3 studies were assessed using two-way ANOVAs (treatment × day) to examine both the main effects, and the interactions between the variables. Variables such as weight and water consumption where data were measured multiple times were analyzed using mixed-model ANOVAs to account for the repeated measures aspect of the study design and included animal as a random factor to account for the correlation structure of the individual animals. In each analysis, data were examined for homogeneity of variance by examining residual values, and by Levene’s test. Some variables were log transformed and reanalyzed where necessary to meet the assumptions of the model. *Post-hoc* comparisons were performed using Dunnett’s test to compare each treatment to the control group in Phases 1 and 2 studies. Phase 3 studies used the Tukey HSD for *post hoc* comparisons. If log transformation failed to correct heterogeneous variance, then nonparametric statistics were utilized including the Kruskal–Wallis test followed by the Steel method to compare each treatment with the control group, or the Steel–Dwass method to compare all treatment groups to one another. Pathology score variables and DAI scores were analyzed using the nonparametric analyses just described. All differences were considered significant at *p* < 0.05.

Cytokine data were visualized using SRPlot (Tang et al. 2023). Concentration data for each cytokine/chemokine for each treatment were uploaded into SRPlot and visualized using bidirectional complete clustering heat maps based on Euclidian distance. Statistical significance between treatment was overlaid on the relevant boxes of the heat maps.

## Results

### Dose response studies

**Figure 2.**
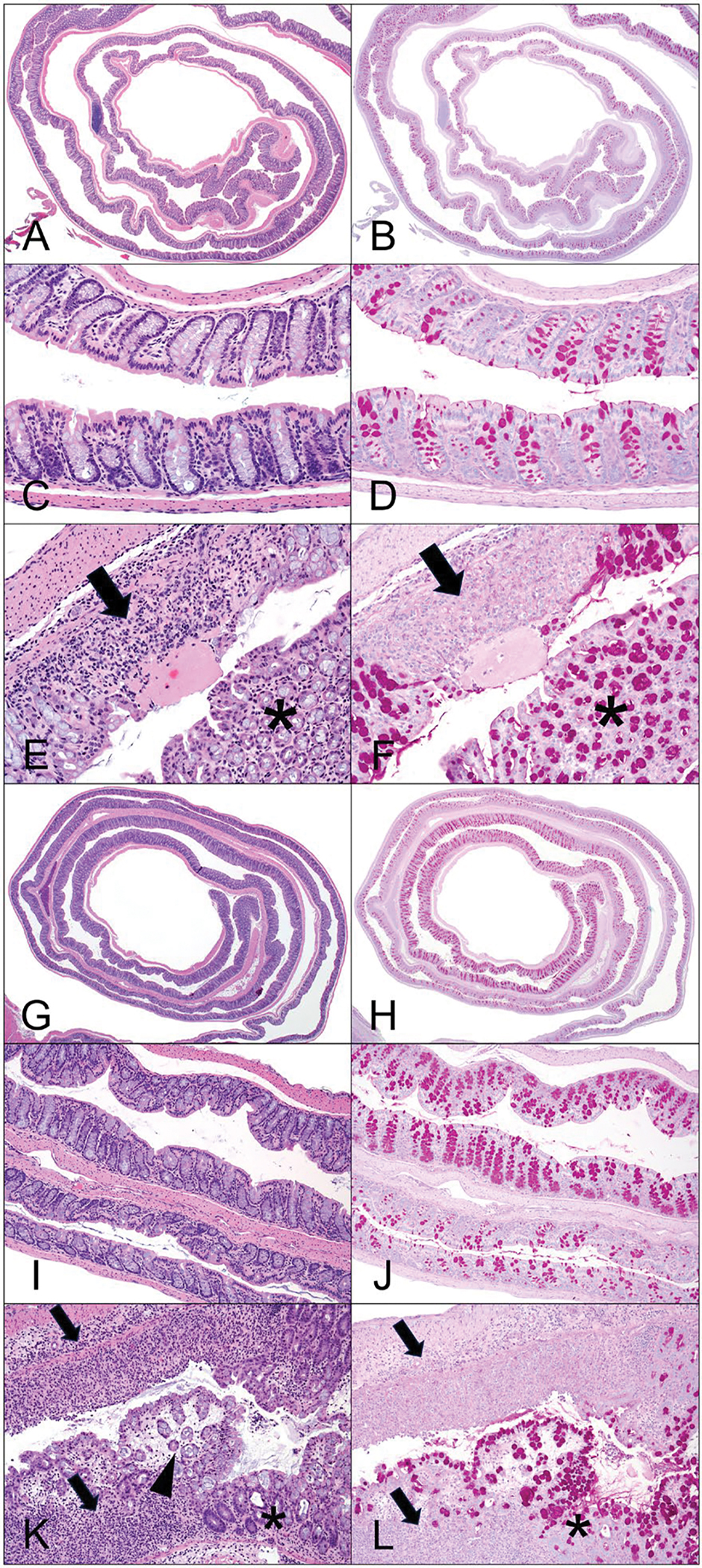
Representative histopathology images of colon from treated (2% DSS) and untreated mice (0% DSS) on regular and HSD. (A–F) Photomicrographs of colon from treated and untreated mice fed regular diet. (G–L) Photomicrographs of colon from treated and untreated mice fed HSD. (A) Normal colon in untreated (0% DSS) mouse fed regular diet, 2X magnification. HE. (B) Normal colon in untreated (0% DSS) mouse fed regular diet, 2X magnification. Periodic acid–Schiff (PAS). PAS staining is used to highlight mucous producing goblet cells within colonic mucosa. (C) Normal colon in untreated mouse fed regular diet, 10X magnification. HE. (D) Normal colon in untreated mouse fed regular diet, 10X magnification. PAS. (E and F) Colon from a treated (2% DSS) mouse fed regular diet, harvest Day 28. (E) Note area of necrosis within the mucosa is ulcerated and infiltrated by large numbers of mixed inflammatory cells (arrow). An area of mucosal hyperplasia, characterized by mucosal thickening and increased numbers of goblet cells is also present (asterisk). 20X magnification, HE. (F) Lack of PAS staining within area of necrosis is consistent with epithelial cell loss (arrow). Intense PAS staining within area of mucosal hyperplasia denotes increased numbers of goblet cells (asterisk). 20X magnification. PAS. (G) Normal colon in untreated (0% DSS) mouse fed high sodium diet, 2X magnification. HE. (H) Normal colon in untreated (0% DSS) mouse fed high sodium diet, 2X magnification. PAS. (I) Normal colon in untreated mouse, 10X magnification. HE. (J) Normal colon in untreated mouse fed high sodium diet, 10X magnification. PAS. (K–L) Colon from a treated (2% DSS) mouse fed high sodium diet, harvest Day 28. (K) Multiple areas of necrosis are present, with large numbers of mixed inflammatory infiltrates (arrows). Areas of mucosal edema (arrowhead) and hyperplasia (asterisk) are also observed. 10X magnification. HE. (L) Lack of PAS staining within area of necrosis is consistent with epithelial cell loss (arrow). Intense PAS staining within area of mucosal hyperplasia denotes increased numbers of goblet cells (asterisk) 10X magnification. PAS.

**Figure 3.**
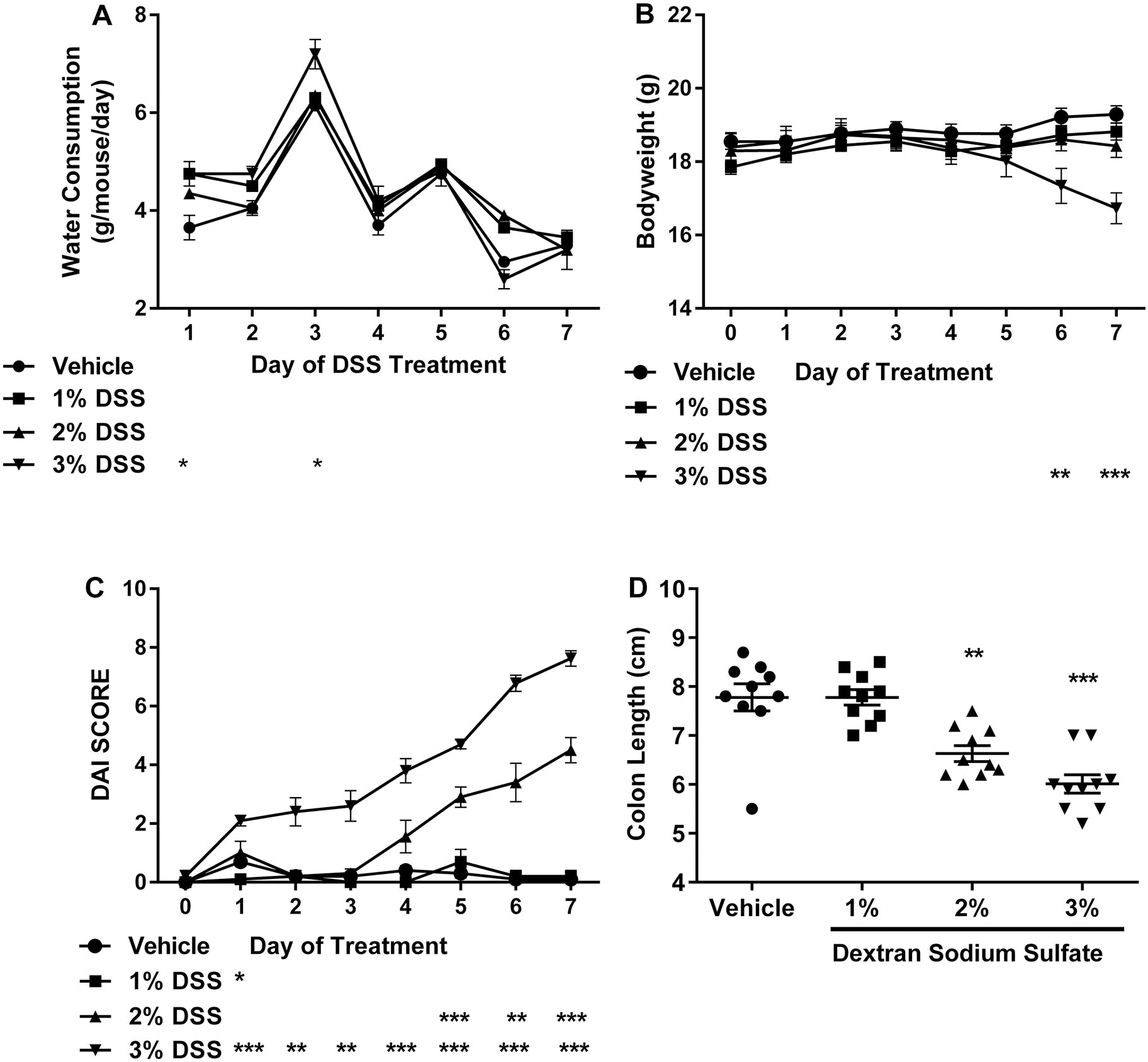
Phase 1: Dose–response assessment of IBD in mice treated with 1%, 2%, or 3% DSS for 7 d: (a) water consumption; (B) bodyweight changes; (C) disease activity index (DAI); (D) colon length. Values represent mean ± SEM. Statistically significant from vehicle control group at *p < 0.05, **p < 0.01, ***p < 0.001.

The study was divided into three phases: dose-response, recovery, and proof of concept using a HSD. The study design for the three phases is shown in Figure 1. The goal of phase 1 was to identify the most appropriate concentration of DSS that produced a clear but modest degree of colon inflammation while still allowing for easy detection of exacerbation caused by test agents. Mice were administered DSS at concentrations of 0%, 1.0%, 2.0%, or 3.0% in drinking water for 7 consecutive days. Histological examination revealed that while 1% DSS did not induce any noteworthy changes in the colon, administering 2% or 3% concentrations were associated with hyperplasia of the mucosal lining, areas of necrosis, mixed inflammation of the lamina propria (inflammatory infiltrates composed of neutrophils, macrophages, and lymphocytes), atrophy of mucosal glands, lymphocyte hyperplasia (increased cellularity of gut-associated lymphatic tissue [GALT]) and glandular dilation (representative micrographs are provided in Figure 2). Scoring indicated that these effects were mild in that none of the lesions affected more than 10% of the tissue (Table 4). The pathology was slightly more prominent in mice that received 3% DSS compared to those that received 2% DSS. The results for drinking water consumption, bodyweights, colon length, and DAI are shown in Figure 3. There was a significant increase in drinking water consumption in mice that received 3% DSS but this was only observed on Days 1 and 3 (Figure 3(A)). Bodyweights were reduced in the 3% DSS treatment group which was significantly different from the vehicle group on Days 6 and 7 of treatment (Figure 3(B)). A progressive increase in the DAI score occurred within several days following the start of administration of 2% or 3% DSS with the 3% treatment group being more severely affected as this group had significantly increased DAI scores within the first 24 h of DSS exposure (Figure 3(C)). The DAI score was not affected in the 1% treatment group other than a minor increase on Day 1 that was not maintained. Associated with the histopathology changes and increased DAI scores, there was a dose-dependent decrease in colon length in both the 2% and 3% DSS treatment groups with the 3% treatment group being more severely affected (Figure 3(D)).

**Figure 4.**
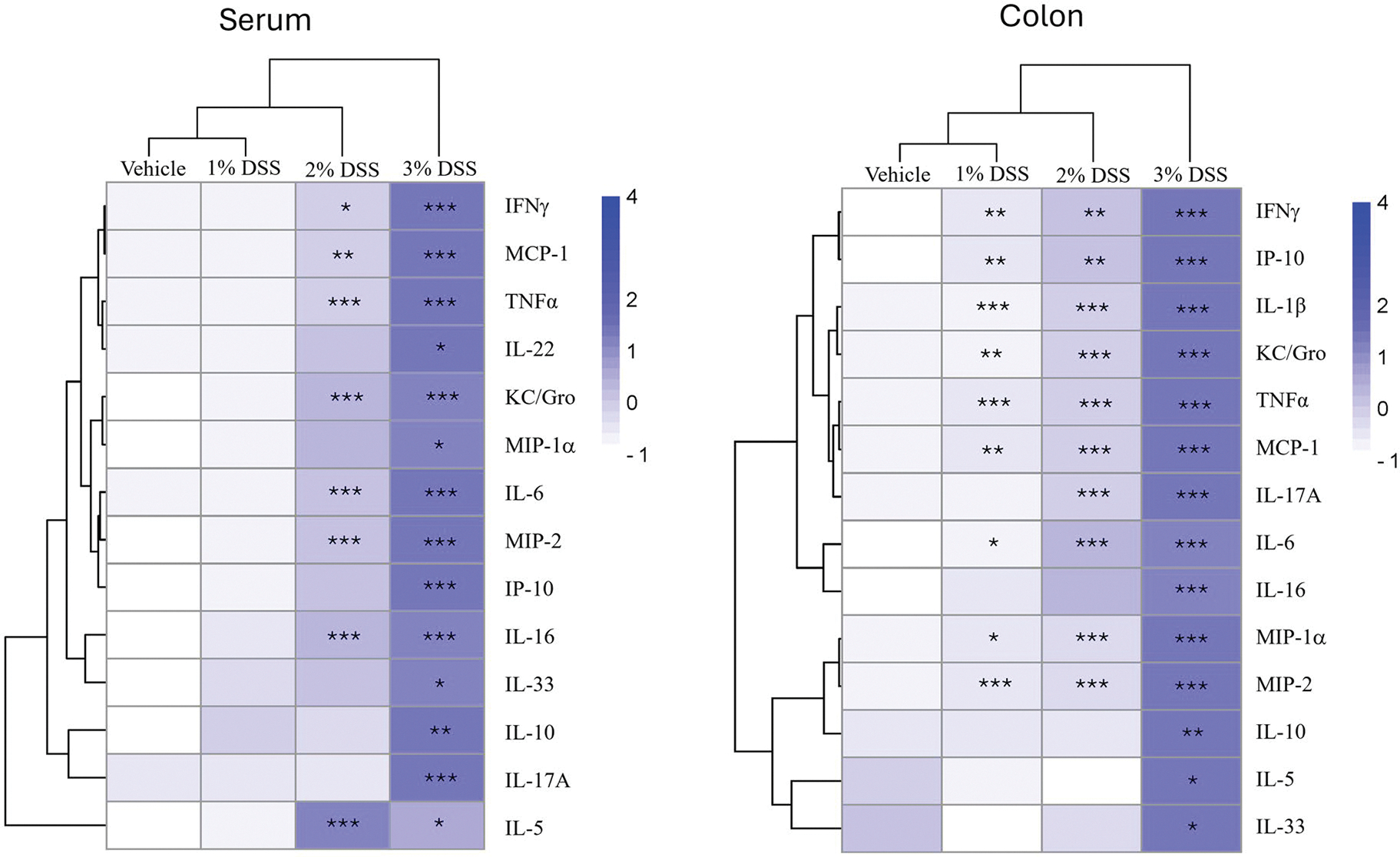
Phase 1: Dose–response assessment of cytokine and chemokine production in serum and colon of mice treated with 1%, 2%, or 3% DSS for 7d. Heat maps represent mean relative changes in cytokine and chemokine levels in the serum and colon with increasing color intensity associated with greater production. Statistically significant from vehicle control group at *p < 0.05, **p < 0.01, ***p < 0.001. Quantitative values of cytokines and chemokines are provided in Supplemental Figures 1–6.

Cytokine and chemokine levels in the colon were measured to understand the immune and inflammatory changes associated with the IBD pathology. In addition, the same cytokines and chemokines were measured in the serum to determine if the immune and inflammatory changes were confined to the colon or were also reflected systemically, which would be important for potential noninvasive biomarker identification. Cytokine levels from serum and colon were measured on Day 7 and visualized using heatmaps (Figure 4). Quantitative cytokine and chemokine levels are provided in Supplemental Figures 1–6. For convenience, cytokines that were not affected by treatment were not included on the maps. Most of the cytokines induced in the colon could also be found in the serum although, as might be expected, their relative levels were lower. While cytokine changes were observed only in the 2% and 3% DSS treatment groups in serum, changes occurred in all DSS treatment groups in the colon, albeit, much lower in the 1% treatment group. The increase in expression of Type 1 pleotropic proinflammatory cytokines (e.g., TNFα, IFNγ, IL-6, IL-17, and IP-10) and chemokines (e.g., MIP-2, KC/GRO, MIP-1α, and MCP-1) predominated in serum and/or colon following DSS treatment. Cytokines known to have anti-inflammatory roles (e.g., IL-5) were minimally induced. These changes are indicative of a pro-inflammatory Type 1 immune response in the colon.

Based upon these dose-response studies which showed a mild degree of IBD in the 2% DSS treatment group compared to the 1% which showed little effect and 3% which produced severe colitis, all further studies were conducted at the 2% concentration.

### Recovery (time course) studies

**Figure 5.**
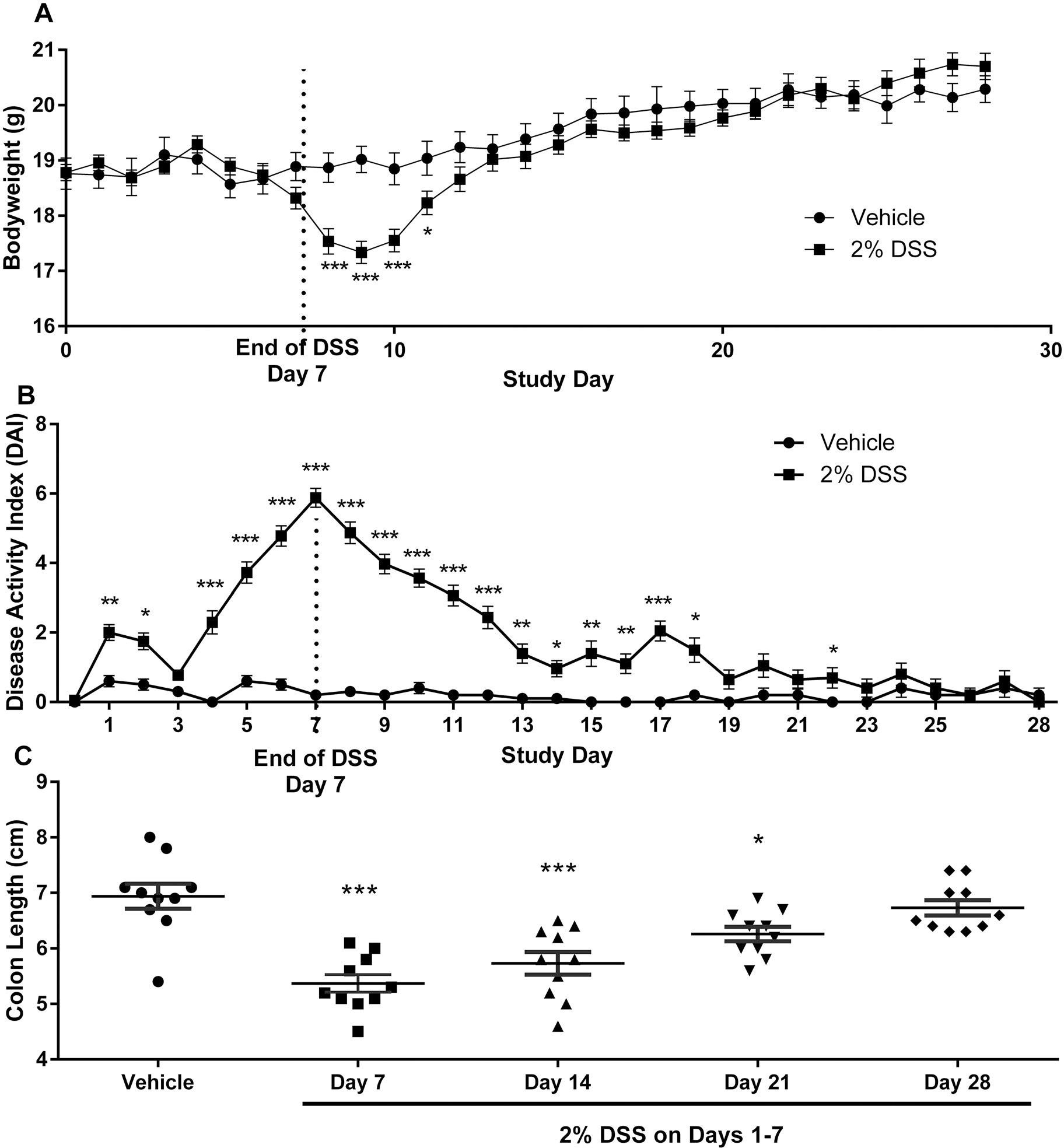
Phase 2: Recovery time course in mice treated with 2% DSS for 7d followed by 21d of recovery without DSS. (A) bodyweight changes; (B) disease activity index (DAI); (C) colon length. Data represent mean ± SEM. Statistically significant from vehicle control group at *p < 0.05, **p < 0.01, ***p < 0.001.

The ability of the colon to return to a normal state was determined over a 21-d monitoring period following administration of 2% DSS for 7 d. The histology severity scores in the colon for markers of inflammation, hyperplasia, and necrosis were highest on Days 7 and 14 with progressive resolution through Days 21 and 28. (Table 5). The severity of these histological changes was mild to moderate and present in the majority of animals (60–100%) on Day 7. By Day 28, the incidence and severity were reduced showing recovery once DSS treatment ceased. Moderate gland dilation was present in 90% of DSS-treated mice on Day 7 and rapidly normalized once DSS was removed with 20% or less of the animals being positive with very low percentage of the colon involved from Day 14 forward. Mucosa atrophy showed a different pattern with progressive increases in incidence as well as colon involvement from Day 7 through the peak of atrophy observed on Days 28. Figure 5 shows the time course for bodyweight changes, the DAI score and colon shortening. Acute effects of DSS were evident as there was a significant reduction in bodyweight on Day 8. The effects of DSS were temporary as bodyweights gradually returned to control values by Day 12 (Figure 5(A)). The DAI score was elevated in DSS-treated animals immediately on Day 1 and peaked on Day 7. Like bodyweight, the effects of DSS on DAI score were temporary as progressive recovery to control values was observed by Day 19 (Figure 5(B)). The maximum effect on colon shortening was also observed on Day 7 in the DSS-treated group before returning to control values by Day 28 (Figure 5(C)). The gross and pathological changes demonstrate that the IBD induced in the present model shows marked recovery within 3 weeks following removal of DSS exposure thereby allowing for detection of environmental agents that alter the course of disease.

**Figure 6.**
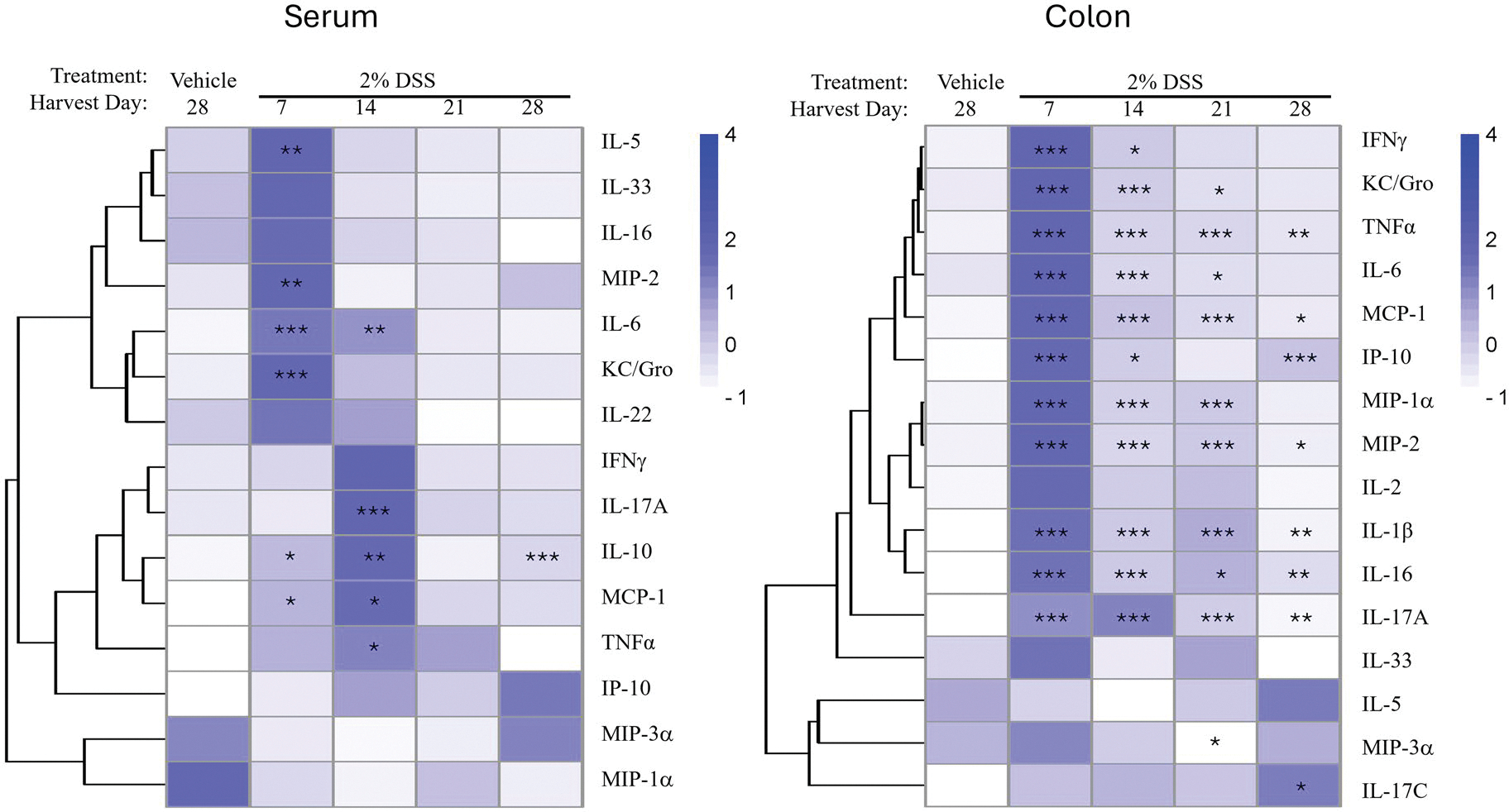
Phase 2: Cytokine and chemokine production in the serum and Colon during the recovery time course in mice treated with 2% DSS for 7d followed by 21d of recovery without DSS. Heat maps represent mean relative changes in cytokine and chemokine levels in the serum and colon with increasing color intensity associated with greater production. Statistically significant from vehicle control group at *p < 0.05, **p < 0.01, ***p < 0.001. Quantitative values of cytokines and chemokines are provided in Supplemental Figures 7–12.

Cytokine levels in the colon and serum also peaked on Day 7 before slowly returning to vehicle-control levels. While several cytokines remained significantly elevated on Day 28 (Figure 6), the magnitude of the change was minor (Supplemental Figures 7–12). Several cytokines, particularly those with regulatory functions, including IL-17A and IL-10, were elevated at later time points and may be involved in resolution of the inflammation and damage.

### Coadministration of high salt diet (HSD)

**Figure 7.**
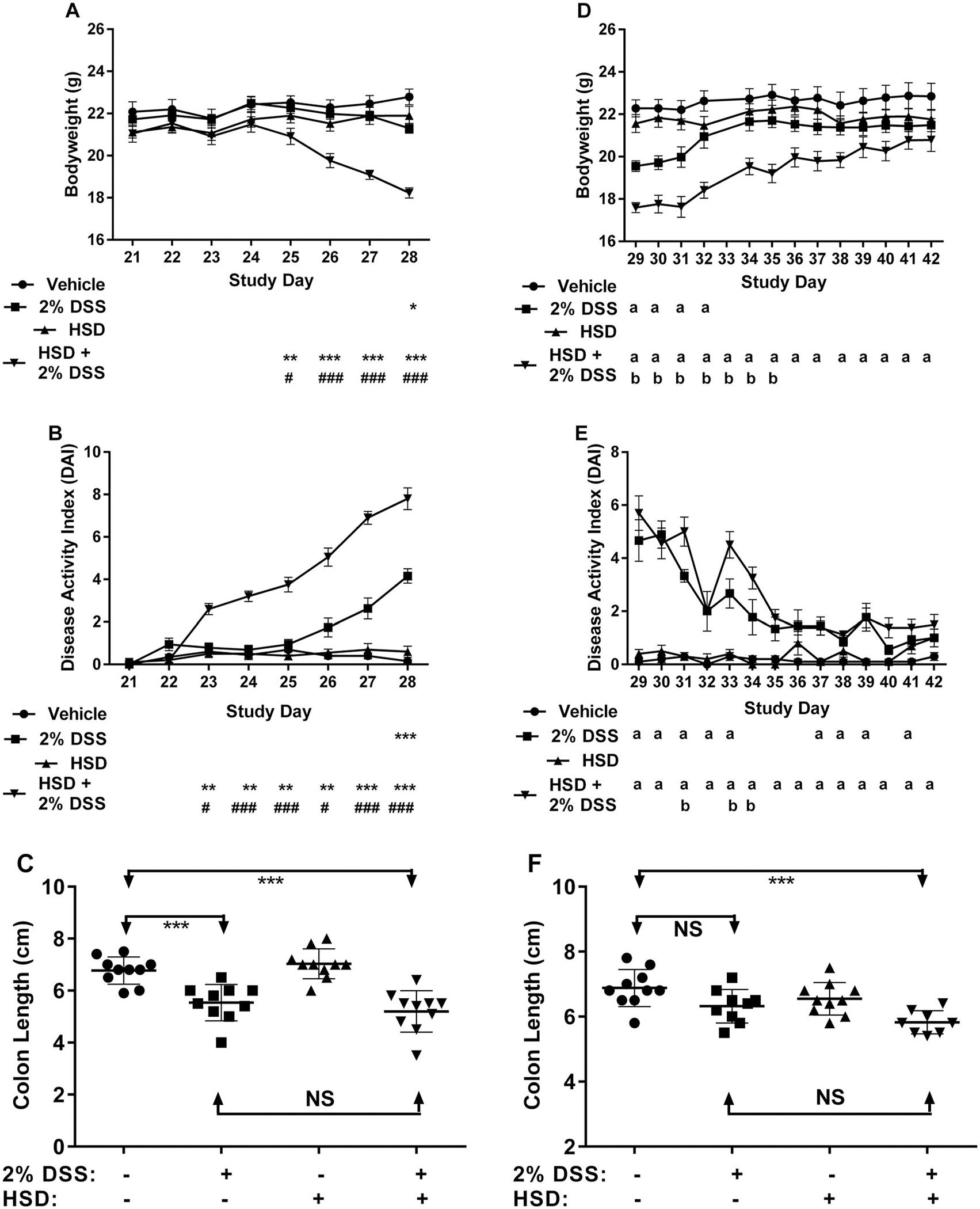
Phase 3: Co-administration of high salt diet (HSD) exacerbated DSS IBD in mice treated with 2% DSS for 7d and impaired recovery following cessation of DSS treatment. Results up to Day 28 at the peak of disease show (A) bodyweight changes; (B) disease activity index (DAI); (C) colon length. Results from Days 29 to 42 during recovery from disease show (D) bodyweight changes; (E) disease activity index (DAI); (F) colon length. Statistically significant from vehicle-control at ^a^*p* < 0.05, *p < 0.05, **p < 0.01. ***p < 0.001. Statistically significant from 2% DSS group at ^b^*p* < 0.05, ^#^*p* < 0.05, ^##^*p* < 0.01, ^###^*p* < 0.001.

The HSD group received NTP-2000 diet supplemented with 4% NaCl and drinking water supplemented with 1% NaCl throughout the study period. Mice were administered DSS for 7 d from Days 21 to 27. Histology scores from animals on the HSD alone were comparable to vehicle controls (Table 6). Elevated histology scores occurred on Day 28 with evidence of resolution for some endpoints by Day 42 in mice exposed to DSS only. Histological changes consisted of hyperplasia, necrosis, atrophy, and inflammation of the colon mucosa. Co-administration of the DSS with HSD exacerbated the pathology in the colon on Day 28, where the incidence and severity of inflammation, edema, and necrosis were elevated compared to mice that received only DSS. Importantly, mice fed the HSD showed less recovery of the DSS-induced histological changes in the colon relative to the DSS-only group as shown on Day 42. Similarly, when compared to mice treated with DSS only, bodyweights following combined HSD and DSS treatment were not only lower during DSS treatment (Figure 7(A)) but remained lower for a longer period (Figure 7(D)). The DAI score was also markedly exacerbated by combined exposure to HSD during the DSS treatment (Figure 7(B)) and showed incomplete recovery compared to DSS-only (Figure 7(E)). Colon shortening in mice exposed to HSD and DSS was similar to mice treated with DSS only after the DSS treatment ended on Day 28 (Figure 7(C)). However, the colon remained shortened in mice exposed to HSD+DSS on Day 42 relative to the vehicle-treated mice, whereas colon length had normalized in the mice receiving DSS only (Figure 7(F)). None of these parameters, including bodyweights, DAI score, or colon length were affected in mice that received only HSD.

Cytokine levels in serum and colon were markedly exacerbated following treatment of mice with HSD and DSS compared to DSS alone at the peak of disease on Day 28 (Figure 8; Supplemental Figures 13–24). Consistent with the pathology and DAI, there was minimal recovery as most cytokines and chemokines that were exacerbated by HSD remained elevated relative to the DSS only and vehicle groups even 2 weeks after the end of DSS treatment.

## Discussion

Oral administration of DSS, a sulfated polysaccharide, in rodents has been used as a model to induce IBD and investigate the effectiveness of various therapeutics on disease progression and pathogenesis (Sann et al. 2013). The DSS-induced IBD model recapitulates many of the hallmark clinical and pathological features associated with IBD in humans including: (1) damage/disruption of the epithelial barrier; (2) alteration of mucus production and composition; (3) alteration of the gut microbiome; and, (4) a shift toward a pro-inflammatory state in the gut. As shown in the current study, this model, with appropriate modification, should be suitable for identifying environmental agents that may accelerate, exacerbate, and/or prolong IBD. The pathology in this model is limited to the colon and characterized by erosions/ulcers, loss of crypts, and infiltration of inflammatory cells. Inflammation primarily occurs in the lamina propria but only in areas underlying the defective epithelium, suggesting that entry of commensal microorganisms into a normal lamina propria may induce an inflammatory response. IBD in humans primarily manifests as colon damage although the entire GI tract can be involved and is known to be a chronic and relapsing disease (Cosnes et al. 2011). Subjects with IBD can experience periods of reduced inflammation and symptoms followed by periods of heightened inflammation which can be refractory to treatment, a finding that may be strongly influenced by macrophage phenotypes (Na et al. 2019). The natural history of IBD is strongly influenced by genetic predisposition but environmental factors are also implicated (Vatn 2009). We also noted in the present study that mild to moderate IBD associated with lower concentrations of DSS (2% w/v in drinking water) is reversible, with most pathological indicators, including histological and microscopic features in the colon, changes in colon length, the DAI and cytokine levels returning to control or near-control values within 3 weeks following cessation of DSS treatment. This is important as it provides an opportunity to assess whether the test agent attenuates recovery of IBD and/or promotes a chronic disease phenotype. Future experiments examining severity of disease following subsequent re-exposure to DSS would extend the potential of the model to show chronic relapsing inflammation as seen in humans.

Some of the advantages of the model include the ability to control the severity and timing/onset of damage to the colon as well as the ability to monitor recovery. Using HSD, we also demonstrated that the model is amenable to discerning the potential of exogenous agents to influence new onset of IBD as well as exacerbate existing disease and prolong/inhibit recovery. While mice that received the HSD only had no colon pathology, co-administration of HSD with DSS exacerbated all pathological and immunological indicators of disease.

Changes in cytokine levels coincided with other indicators of colon pathology that were measured following DSS treatment with peak levels seen on Day 7, after the last day of DSS administration, followed by a moderately slow return for most cytokines to control levels by Day 28. Cytokine levels were markedly exacerbated in DSS treated mice that were on the HSD with most of the cytokine and chemokines remaining highly elevated on Day 42, 2 weeks after cessation of DSS treatment. Since Day 42 was the last time point examined, we were not able to determine whether the combined exposure induced a temporary delay in recovery or transformed it into a chronic disorder. The HSD given alone had no effect on cytokine expression supporting a synergistic effect of coadministration of the exogenous agent on the effects of DSS.

The cytokine profiles provided additional data on the pathological nature of the response. For the most part the profiles detected in serum and from colon extracts were similar although, as might be expected, the latter had relatively higher levels being the site of damage. There were several exceptions such as IFNγ, which was elevated in the colon but not serum following administered 2% DSS but was present in serum in mice administered 3% DSS. This suggests that serum cytokine/chemokine production is dependent on the extent of damage to the colon. IFNγ, which is primarily produced by CD4^+^ T- helper 1 (TH1) cells, natural killer (NK) cells, and CD8^+^ cytotoxic T-cells, is a major pathological driving force of UC (Neurath 2014; Leppkes and Neurath 2020). In contrast, IL-22, was elevated in serum but was not present in the colon following DSS treatment. The role of IL-22 in IBD etiopathogenesis is less clear as it has been implicated in both the early development as well as the late resolution of UC (Zenewicz et al. 2008; Zhao et al. 2023). There was limited evidence that cytokines involved in TH1 or TH2 cell regulation were altered although IL-5, which is produced by TH2 cells and mast cells, was elevated in the serum and to a lesser extent in the colon after DSS treatment. In fact, IL-5 remained elevated during the recovery phase suggesting that it may be involved in reduction of the inflammation and repair of the colon, consistent with the known intestinal homeostatic and repair roles for Type 2 immune responses (Luo and Villablanca 2021). However, the role of IL-5 may be more complicated as recent evidence suggests that IL-5 plays a role in DSS-induced activation of the NLRP3 inflammasome and chemical antagonism of the IL-5 receptor ameliorated DSS-induced IBD (Ou et al. 2024). Elevated IL-10 was also noteworthy as it is considered an anti-inflammatory cytokine (Eskdale et al. 1997), and IL-10 deficiency (Berg et al. 1996) or neutralization (Saha et al. 2021) in mice can result in rapid and progressive colon inflammation. Mutations in IL-10 and its receptor have been identified as risk factors for development of very early onset IBD in humans (Zhu et al. 2017). Given the inverse relationship between IL-10 and IBD, it is possible that elevation of IL-10 in the present DSS IBD model may represent a compensatory anti-inflammatory response to the damage. IL-10 was significantly elevated in severe IBD induced by 3% DSS treatment, however, it was not significantly increased in less severe disease following 2% DSS treatment. However, IL-10 production significantly increased in the recovery phase following cessation of DSS treatment supporting the potential for anti-inflammatory compensation. Consistent with these findings, the therapeutic mechanism of anti-TNFα treatment for IBD has been linked to repolarization of proinflammatory macrophages to CD206+ regulatory macrophages with increased IL-10 production (Koelink et al. 2020).

Several Type 1 cytokines, including IL-33 and IL-17A, that were elevated in DSS-treated mice in these studies are also known to be elevated in the blood and inflamed mucosa of patients with active IBD. Both IL-33 (Ajduković et al. 2010; Beltrán et al. 2010) and IL-17A (Fujino et al. 2003) have been identified as biomarkers of severity and potential therapeutic targets for IBD (Majeed and Motaweq 2025; Nardone et al. 2025). IL-33 is a nuclear cytokine from the IL-1 family that plays a critical role in initiating innate immune responses (Cayrol and Girard 2018, 2014; Cayrol 2021). Its role in IBD is unclear but may involve the differentiation of enterocytes into goblet cells and restoration of intestinal mucus production leading to improvement in IBD symptoms in DSS-treated mice, effects that may be mediated through IL-33-induced M2 polarization of macrophages (Seo et al. 2017). Elevated IL-17A observed in the present model is also noteworthy as IL-17 inhibitors have been used to treat patients with CD, although with mixed success (Hueber et al. 2012; Targan et al. 2016). IL-17A, which links T cell activation to neutrophil activation and mobilization, is driven in mice by IL-6 and TGFβ (Zenobia and Hajishengallis 2015) and is known to be a key cytokine in the pathogenesis of IBD. The presence of elevated IL-17A and IL-33 in the serum of DSS-treated mice support the role for cytokines as potential biomarkers of IBD severity and exacerbation.

In summary, these studies demonstrate that the present DSS model is suitable to identify potential environmental agents that can accelerate or exacerbate IBD or interfere with disease recovery. In addition to chemically induced animal models for IBD, there are models which employ adoptive T- cell transfer or genetic modification in rodents (Maxwell and Viney 2009). While relevant in drug discovery and basic research studies, such models are not well-suited for screening/identification of environmental factors as the exogenous agents would have limited ability to influence disease onset, severity or recovery. One disadvantage of the DSS model is the uncertainty of the role played by autoimmune pathogenic mechanisms. However, it is apparent that data favoring classical autoimmune pathogenic mechanisms, like antigen-specific autoreactive T- or B-cells, are scant and not necessarily robust in human IBD (Wen and Fiocchi 2004). Nevertheless, the present DSS model, exhibits clear evidence that IBD is an inflammatory disease, with results that align with an immune and inflammatory etiology.

## Supplementary Material

Supp 1

Supplemental data for this article can be accessed online at https://doi.org/10.1080/1547691X.2025.2594800.

## Figures and Tables

**Figure 8.**
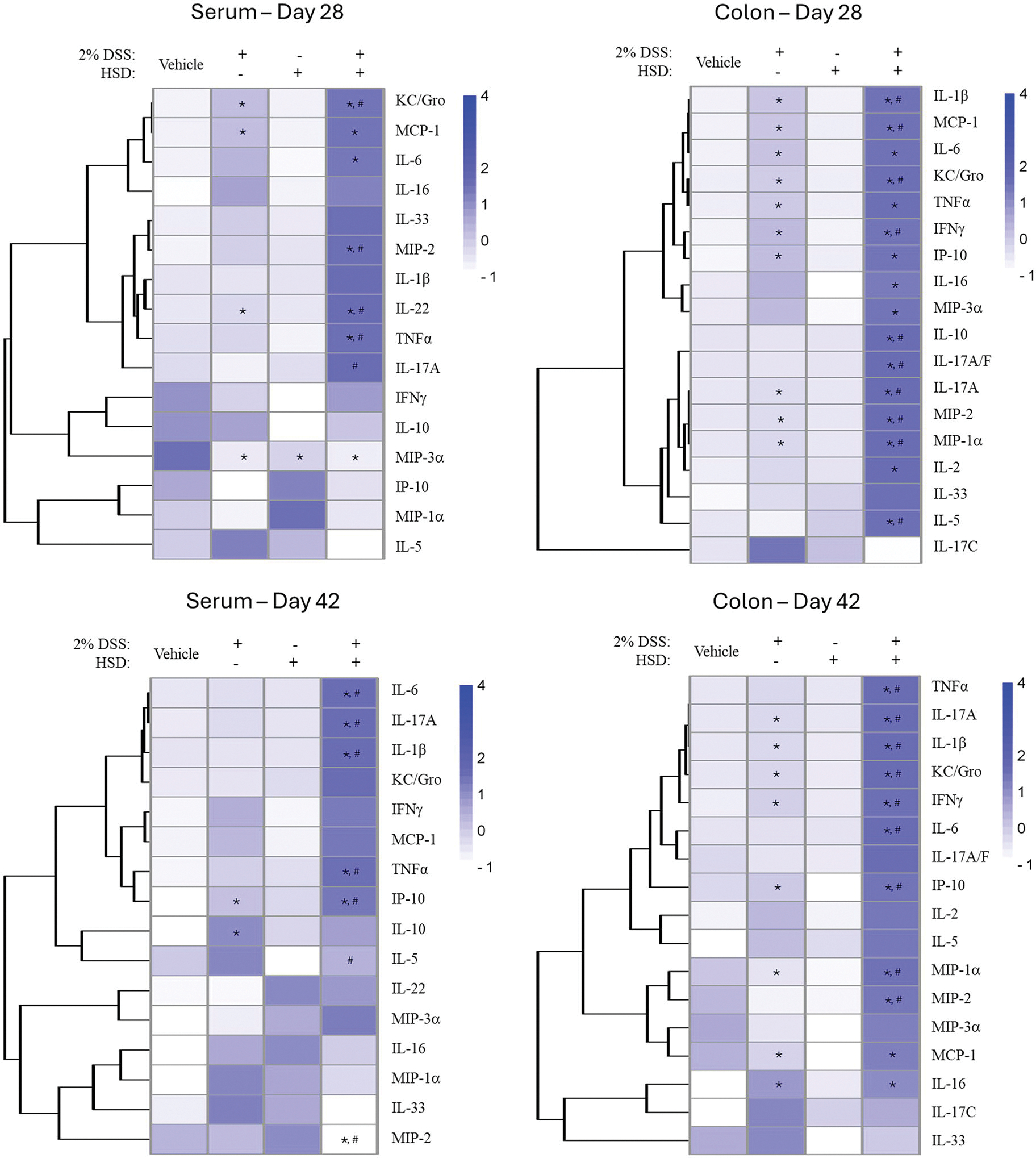
Phase 3: Co-administration of high salt diet (HSD) exacerbated cytokine and chemokine production in the serum and colon of mice treated with 2% DSS for 7d and impaired recovery following cessation of DSS treatment. Groups of mice received vehicle control, continuous HSD, 2% DSS for 7d, or continuous HSD plus 2%DSS for the last 7d. Heat maps represent mean relative changes in cytokine and chemokine levels in the serum and colon with increasing color intensity associated with greater production. Statistically significant from vehicle control group at *p < 0.05, **p < 0.01, ***p < 0.001. Statistically significant from 2% DSS group at ^#^*p* < 0.05. Quantitative values of cytokines and chemokines are provided in Supplemental Figures 13–24.

**Table 1.** Scoring for disease activity index (DAI*) in mice administered DSS.

	Score				
	
Parameters assessed	0	1	2	3	4

Weight loss	None	<10%	10–15%	15–20%	>20%
Stool consistency	Normal	–	Loose	–	Diarrhea
Blood in stool	Negative	–	Positive	–	Gross bleeding

* DAI for each individual animal is the sum of the scores for weight loss (0–4), stool consistency (0–4), and the presence of occult blood in stool (0–4). The range for DAI is 0 (no indications of colitis) to 12 (severe colitis).

**Table 2.** Histological features of Colon mucosa in DSS-treated mice.

Histological feature	Brief description

Colon, mucosa: Hyperplasia	Thickening of the lamina propria (mucosal lining) of the colon with areas of increased cellularity, cell crowding and piling of epithelial cells. Often indicative of a regenerative change following some degree of epithelial damage.
Colon, mucosa: Necrosis	Loss or damage of the lamina propria. Normal tissue architecture is replaced with variable amounts of eosinophilic, proteinaceous and cellular debris. Associated with areas of inflammation.
Colon, mucosa: Inflammation, mixed	In most instances, included edema of the lamina propria and underlying submucosa with dilated mucus glands, and inflammatory infiltrates consisting mainly of neutrophils with fewer macrophages and lymphocytes.
Colon, mucosa: Atrophy	Decreased mucosal height and depth of mucosal glands often accompanied by reduced numbers of goblet cells. Atrophy often arises secondary to intestinal dilation. Can be difficult to distinguish from artifactual stretching of the tissue.
Colon: Hyperplasia, Lymphocyte	Increased size, number and/or cellularity of lymphoid structures within colonic submucosa.
Colon, gland: Dilation	Enlargement or expansion (dilation) of mucus glands.
Colon: Dilation	Increased space (dilation) within the colon lumen.

**Table 3.** Cytokines/chemokines examined in serum and Colon extracts.

IFN-γ	IL-4	IL-16	IL-22	IL-15	IP-10
IL-10	IL-5	IL-17A	IL-23	IL-17A/F	MCP-1
IL-12p70	IL-6	IL-17C	IL-31	IL-27	MIP-1α
IL-1β	KC/GRO	IL-17F	IL-17E	IL-33	MIP-2
IL-2	TNF-α	IL-21	MIP-3α	IL-9	

**Table 4.** Colon mucosa pathology scores in mice receiving DSS in drinking water.

		DSS concentration (% in drinking water)		
	
	Vehicle	1.00%	2.00%	3.00%

Hyperplasia	0 (0.0)^a^	0 (0.0)	50 (0.5)*	50 (0.5)*
Necrosis	0 (0.0)	0 (0.0)	0 (0.0)	50 (0.5)*
Inflammation, mixed	0 (0.0)	0 (0.0)	90 (0.9)***	100 (1.0)***
atrophy	0 (0.0)	0 (0.0)	70 (0.8)**	50 (0.5)*
Gland dilation	0 (0.0)	0 (0.0)	0 (0.0)	0 (0.0)

a Data are presented as % of animals affected (average pathology score). DSS – dextran sodium sulfate. DSS was provided in the drinking water Days 0–6 (7 d of exposure) and terminal necropsy was performed on Day 7. Pathology scoring: 0 = no lesion/within normal limits; 1 = up to 10% of the tissue affected; 2 = 10–25% of the tissue affected; 3 = 25–50% of the tissue affected; 4 = >50% of the tissue affected. Average severity rounded to nearest 10th. Ten animals were used per treatment group.

Statistically significant from vehicle control group at *p < 0.05, **p < 0.01, ***p < 0.001.

**Table 5.** Colon mucosal pathology scores over a 28 Day period (Phase 2 recovery studies).

		DSS 2% recovery timepoint			
	
	Vehicle	Day 7	Day 14	Day 21	Day 28

Hyperplasia	0 (0.0)	100 (1.4)***	90 (1.1)***	80 (1.3)**	60 (0.6)*
Necrosis	0 (0.0)	60 (0.9)*	100 (1.3)***	30 (0.5)	30 (0.4)
Inflammation, mixed	0 (0.0)	100 (1.5)***	100 (1.7)***	90 (1.3)***	70 (0.9)**
Atrophy	0 (0.0)	70 (0.8)**	50 (0.5)*	90 (1.1)***	100 (1.5)***
Hyperplasia–lymphocyte	0 (0.0)	0 (0.0)	30 (0.3)	20 (0.2)	40 (0.4)
Gland Dilation	0 (0.0)	90 (1.2)***	20 (0.2)	10 (0.1)	20 (0.2)

Dextran sulfate (DSS) was provided in the drinking water Days 0–6 (7 d of exposure) and terminal necropsy was performed on the days indicated. To reduce the number of animals used, a single vehicle group was euthanized on day 28. Scoring: 0 = no lesion/within normal limits; 1 = up to 10% of the tissue affected; 2 = 10–25% of the tissue affected; 3 = 25–50% of the tissue affected; 4 = >50% of the tissue affected. Average severity rounded to nearest 10^th^. Ten animals were used per treatment group.

Statistically significant from vehicle control group at *p < 0.05, **p < 0.01, ***p < 0.001.

**Table 6.** Colon mucosal pathology scores on Days 28 and 42 in mice treated with DSS and fed a HSD.

	Day 28				Day 42		
		
	Vehicle	2% DSS	HSD	2% DSS and HSD	2% DSS	HSD	2% DSS & HSD
		
Mice (N)	10	10	10	10	9	10	8

Hyperplasia	0 (0.0)	90 (1.3)***	0 (0.0)	90 (1.2)***	78 (0.9)**	0 (0.0)	88 (1.3)**
Necrosis	0 (0.0)	50 (0.6)*	0 (0.0)	70 (1.2)**	67 (0.7)**	0 (0.0)	88 (1.1)**
Inflammation-mixed	0 (0.0)	60 (0.7)*	0 (0.0)	100 (1.4)***	67 (0.8)**	0 (0.0)	88 (1.3)**
Edema	0 (0.0)	10 (0.1)	0 (0.0)	30 (0.4)	0 (0.0)	0 (0.0)	13 (0.1)
Atrophy	0 (0.0)	60 (0.9)*	0 (0.0)	40 (0.4)	11 (0.1)	0 (0.0)	75 (0.8)**^,#^
Hyperplasia–lymphocyte	0 (0.0)	0 (0.0)	0 (0.0)	10 (0.1)	56 (0.6)	0 (0.0)	63 (0.6)*
Gland-dilation	0 (0.0)	10 (0.1)	0 (0.0)	10 (0.1)	11 (0.1)	10 (0.1)	25 (0.3)

Dextran sodium sulfate (DSS) was provided in the drinking water Days 21–27 (7 days of exposure) and high salt diet (HSD) was provided continuously (0–42) in feed/ Terminal necropsy was performed on the days indicated. To reduce the number of animals used, a single vehicle group was euthanized on Day 28. Scoring: 0 = no lesion/within normal limits; 1 = up to 10% of the tissue affected; 2 = 10–25% of the tissue affected; 3 = 25–50% of the tissue affected; 4 = >50% of the tissue affected. Data are presented as percent incidence (average score). Severity rounded to nearest 10th. N = number of mice per treatment group.

Statistically significant from vehicle control group at *p < 0.05, **p < 0.01, ***p < 0.001.

Statistically significant from 2% DSS group at ^#^*p* < 0.05.
