# Supplementary material for: Modification of the dextran sodium sulfate model to identify agents that induce or exacerbate inflammatory bowel disease
Source: J Immunotoxicol. Author manuscript; Available in PMC 2026 Feb 7. (PMC12882113; doi:10.1080/1547691X.2025.2594800)
Supplement: Supp 1 [file NIHMS2134587-supplement-Supp_1.docx]

**Modification of the dextran sodium sulfate model to identify agents that induce or exacerbate inflammatory bowel disease**

Victor J. Johnson^1*^, Michael I. Luster^1^, Michael Kashon^2^, Erin M. Quist^3,a^, Gary R. Burleson^1^, Florence G. Burleson^1^, Dori R. Germolec^4^

^1^Burleson Research Technologies, Inc., Morrisville, NC, USA

^2^Mountainview Research Analytics, LLC., Morgantown, WV, USA

^3^Experimental Pathology Laboratories, Inc., Durham, NC, USA

^4^Division of Translational Toxicology, National Institute of Environmental Health Sciences, NIH, Research Triangle Park, NC, USA

**Supplemental Figures**

Phase 1 – Cytokine and Chemokine Concentrations in Serum and Colon

Supplemental Figure 1

Supplemental Figure 2

Supplemental Figure 3

Supplemental Figure 4

Supplemental Figure 5

Supplemental Figure 6

Phase 2 Cytokine and Chemokine Concentrations in Serum and Colon

Supplemental Figure 7

Supplemental Figure 8

Supplemental Figure 9

Supplemental Figure 10

Supplemental Figure 11

Supplemental Figure 12

Phase 3: Serum and colon cytokine/chemokine production

Supplemental Figure 13

Supplemental Figure 14

Supplemental Figure 15

Supplemental Figure 16

Supplemental Figure 17

Supplemental Figure 18

Supplemental Figure 19

Supplemental Figure 20

Supplemental Figure 21

Supplemental Figure 22

Supplemental Figure 23

Supplemental Figure 24
